# Development of an Immune Infiltration-Related Prognostic Scoring System Based on the Genomic Landscape Analysis of Glioblastoma Multiforme

**DOI:** 10.3389/fonc.2020.00154

**Published:** 2020-02-18

**Authors:** Guihua Tang, Wen Yin

**Affiliations:** ^1^Department of Clinical Laboratory, Hunan Provincial People's Hospital (The First Affiliated Hospital of Hunan Normal University, The College of Clinical Medicine of Human Normal University), Changsha, China; ^2^Department of Neurosurgery, Xiangya Hospital of Central South University, Changsha, China

**Keywords:** glioblastoma multiforme (GBM), immune infiltration, prognostic scoring system, TCGA, CGGA

## Abstract

**Introduction:** Glioblastoma multiforme (GBM) is the most common deadly brain malignancy and lacks effective therapies. Immunotherapy acts as a promising novel strategy, but not for all GBM patients. Therefore, classifying these patients into different prognostic groups is urgent for better personalized management.

**Materials and Methods:** The Cell type Identification by Estimating Relative Subsets of RNA Transcripts (CIBERSORT) algorithm was used to estimate the fraction of 22 types of immune-infiltrating cells, and least absolute shrinkage and selection operator (LASSO) Cox regression analysis was performed to construct an immune infiltration-related prognostic scoring system (IIRPSS). Additionally, a quantitative predicting survival nomogram was also established based on the immune risk score (IRS) derived from the IIRPSS. Moreover, we also preliminarily explored the differences in the immune microenvironment between different prognostic groups.

**Results:** There was a total of 310 appropriate GBM samples (239 from TCGA and 71 from CGGA) included in further analyses after CIBERSORT filtering and data processing. The IIRPSS consisting of 17 types of immune cell fractions was constructed in TCGA cohort, the patients were successfully classified into different prognostic groups based on their immune risk score (*p* = 1e-10). What's more, the prognostic performance of the IIRPSS was validated in CGGA cohort (*p* = 0.005). The nomogram also showed a superior predicting value. (The predicting AUC for 1-, 2-, and 3-year were 0.754, 0.813, and 0.871, respectively). The immune microenvironment analyses reflected a significant immune response and a higher immune checkpoint expression in high-risk immune group.

**Conclusion:** Our study constructed an IIRPSS, which maybe valuable to help clinicians select candidates most likely to benefit from immunological checkpoint inhibitors (ICIs) and laid the foundation for further improving personalized immunotherapy in patients with GBM.

## Introduction

Glioblastoma multiforme (GBM) is the most common deadly brain malignancy, with an incidence of 3.19 per million person (1) and representing ~1% of new tumor cases worldwide (2), which is indeed characterized by a remarkably poor prognosis, presenting only a 2-year survival rate of 26–33% and a 5-year survival rate of 4–5% (1). The treatment of GBM is of great challenge, and maximal safe surgical resection is served as the first-line treatment (3) to relieve clinical symptoms, prolong survival time and contribute to the pathological diagnosis. Unfortunately, infiltrative cells always exist and prevent complete resection of the surgery which ultimately leads to recurrence, resistance, and death (4). Therefore, the current standard therapy for newly diagnosed GBM is surgical removal followed by concurrent chemoradiotherapy and adjuvant chemotherapy with temozolomide (5). Disappointedly, the median overall survival improves limited and remains around 15 months (1, 5) despite continued innovations in neurosurgical techniques, improvements in radiotherapy, and the emergence of novel chemotherapeutic agents over the past three decades (6). Facing these discouraging situations, advancement of novel strategies in GBM management is warranted.

Immunotherapy has recently gained more and more attention on fighting against tumors and made a great contribution in treating diverse hard-to-treat tumors such as non-small cell lung cancer and melanoma (7, 8), which contributed to extending this therapeutic concept to GBM. However, its clinical efficacy in GBM remains to be further elucidated. The unique blood-brain barrier (BBB) makes the brain a relatively immune privileged organ (2, 9) which induces tightly regulated immune responses and leads to a so-called immunosuppressive microenvironment. Consistently, immunosuppressive microenvironment ubiquitously exists in GBM that involves tumor intrinsic and extrinsic components (10), which leads to a unique challenge in the treatment of this cancer. GBM has a great degree of heterogeneity with intra- and inter-tumoral (10, 11), leading to only a subset of the treated patients benefit from the most successful immunotherapy. This suggests the need for searching novel targets that might be conducive to better stratify patients with different prognosis and identify the candidates who will really benefit from immunotherapy. Because of the establishment of an immunosuppressive microenvironment, understanding the immune landscape, and changing immunosuppressive microenvironment seems a reasonable strategy for GBM. Tumor-infiltrating immune cells have been reported to correlate with clinical prognosis in various tumors (8, 12, 13) and affect the clinical response to immunotherapy in GBM (14), indicating that immune-infiltrating cells may be a promising biomarkers repository for better personalized management of GBM.

Recently, a newly proposed computational algorithm, known as “Cell type Identification By Estimating Relative Subsets Of RNA Transcripts (CIBERSORT),” was developed (15) and successfully applied to analyse the immune cell types in several malignant tumors like colorectal cancer (12), gastric cancer (13), and renal cell carcinoma (16). Therefore, in the present study, we employed CIBERSORT, for the first time, to determine the relative fractions of 22 immune cell subsets in GBM using gene expression profiles. Subsequently, we used the least absolute shrinkage and selection operator (LASSO) Cox regression analysis to construct an immune infiltration-related prognostic scoring system (IIRPSS), classifying patients into different prognostic subgroups based on their IRS. We further explored the relationships between the immune risk groups, the immune infiltration cells, and immune checkpoint modulators. Meanwhile, the immune statuses of different immune risk groups were explored as well by gene set enrichment analysis (GSEA). It is hoped that this study will provide promising targets and some novel insights into the immunotherapy of GBM.

## Materials and Methods

### Patients and Datasets

The Cancer Genome Atlas (TCGA) GBM normalized gene expression array (*n* = 539) from the Affymetrix HT Human Genome U133a microarray platform and relevant clinical information were obtained from the UCSC Xena website (https://xena.ucsc.edu/). Another part of GBM mRNA-Seq data measured using Illumina HiSeq 4,000 and corresponding clinical data were downloaded from the Chinese Glioma Genome Atlas (CGGA) (http://www.cgga.org.cn/index.jsp).

### Data Processing

We extracted 132 primary GBM samples in batch1 and 82 samples in batch2 from CGGA acquired data, respectively. These patients were all with survival data and their survival time was more than 30 days. Afterward, the two mRNA-Seq profiles were separately normalized by the log_2_(x+1) method.

### Cibersort Estimation

CIBERSORT, a deconvolution algorithm based on normalized gene expression profiles, has been validated by fluorescence-activated cell sorting (FACS), which can be used to characterize 22 types of immune infiltration cell composition of complex samples (16). Each gene expression series was separately uploaded to the CIBERSORT web tool (https://cibersort.stanford.edu/), and a reference LM22 expression signature with 100 permutations was used for the algorithm. CIBERSORT, using Monte Carlo sampling, derives a deconvolution *p*-value for each sample. The results of the predicted infiltrating immune cell fractions with an appropriate *p* < 0.05 were considered to be accurate, which were eligible for further analysis. For each sample, all the output estimates of each immune cell type were normalized to sum up to 1 (13), therefore, the annotated cell fraction can be directly compared between different immune cell subsets and platforms (17).

### Construction an Immune Infiltration-Related Prognostic Scoring System

Patients with a CIBERSORT *p* ≥·05 were eliminated in the subsequent analysis, as were those in TCGA dataset with normal or recurrent samples and patients whose overall survival was lacking or no more than 30 days. For the purpose of constructing this scoring system, TCGA dataset played the role as the training set and CGGA as the validation set. Moreover, the estimated cell fraction was served as binary variables, and was given a specific value of 1 or 2 if lower or higher than the optimal cut-off values which were determined for the entire cohort by the web portal “Cutoff Finder” (http://molpath.charite.de/cutoff/) and were calculated with survival: significance (log-rank test) method.

LASSO Cox analysis, as a wildly used high-dimensional predictor regression method (18), selecting the optimal penalty parameter lambda using 10-fold cross-validations to prevent overfitting (19), can achieve shrinkage and variable identify simultaneously (20), and thus, which is an appropriate solution to establish signatures if there are numerous correlated covariates (21). Therefore, we utilized LASSO Cox regression analysis in the training set to establish an IIRPSS by a linear combination of selected prognostic cell compositions among 22 immune cell types weighted by the optimal coefficients. Simultaneously, the prognostic prediction power of this IIRPSS was further validated in the CGGA cohorts. Additionally, the immune risk score (IRS) was demonstrated an independent prognosis factor by univariate and multivariate Cox regression in training as well as the validation set.

### Gene Set Enrichment Analysis

GSEA was performed in TCGA cohort to investigate the potential immune status between high-risk and low-risk phenotypes based on immune-related gene ontology gene sets downloaded from the Molecular Signatures Database (MSigDB, http://software.broadinstitute.org/gsea/msigdb/index.jsp). The significant cutoff value was defined as the false discovery rate (FDR < 0.25) and the nominal *p* < 0.05.

### Statistical Analysis

All the statistical analyses were carried out using R software (version 3.5.1) and considered significant when corresponding *p* < 0.05. The LASSO method was used to establish Cox proportional hazard models, and the Kaplan–Meier (K-M) analysis was implemented to calculate survival rates using the log-rank test. To quantitatively predict the probability of survival, a nomogram was conducted. The time-dependent receiver operating characteristic (ROC) curves were applied to assess the performance of the predictive nomogram, determined by the area under the ROC curve (AUC), and relevant calibration plots were also visualized. The levels of immune cell fractions between groups were assessed by Wilcoxon's Sign Rank Test and displayed with the violin plot. The correlations between the IRS and immune checkpoints expression levels were evaluated by Pearson's correlation test.

## Results

### Patient Characteristics

There were 239 GBM samples from TCGA and 71 samples from CGGA with eligible survival information available for further analyses after appropriately CIBERSORT filtering. The details of patients' characteristics are summarized in Table 1.

**Table 1:** Summary of patient's clinical characteristics.

**Characteristic**		**TCGA set**	**CGGA set**
Age	≤45	52	23
	>45	187	48
Gender	Male	157	41
	Female	82	30
IDH	Wildtype	98	54
	Mutant	13	14
	NOS	128	3

*NOS was defined as a diagnosis for whom IDH status has not been fully evaluated*.

### IIRPSS Construction and Validation

**Figure 1:**
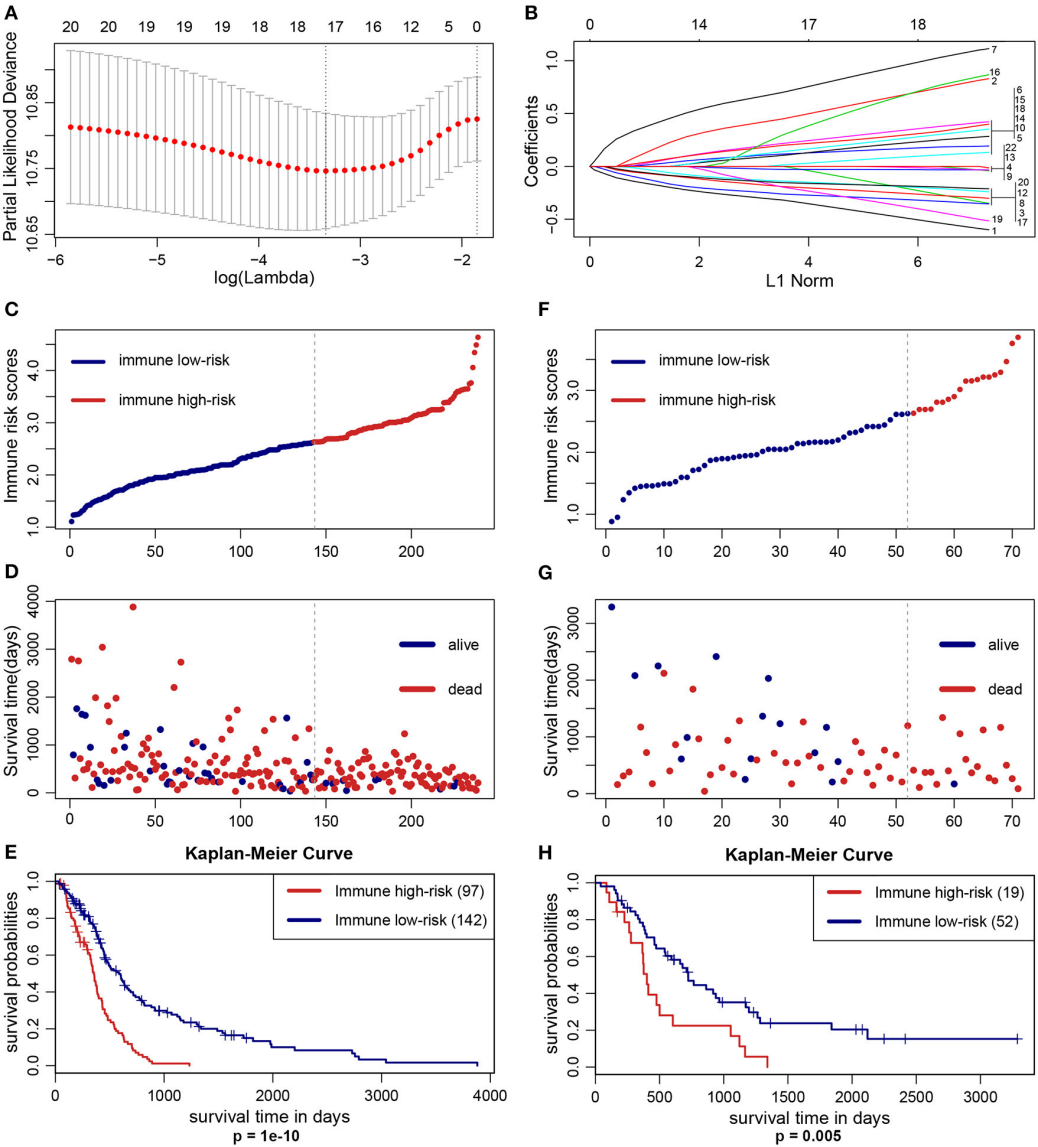
Construction and validation of the IIRPSS. **(A,B)** 17 types of immune cells selected by LASSO Cox regression analysis. Left: using 10-fold cross-validation to the optimal penalty parameter lambda. Right: LASSO coefficient profiles of the immune-infiltrating cells. **(C–E)** Distribution of the IIRPSS in the training (TCGA) cohort. Upper panel: classification of patients into different immune risk groups based on the optimal IRS. Middle panel: distribution of patients' survival time and status. Bottom panel: Kaplan–Meier survival curves between immune low- and high-risk groups. **(F–H)** Validation of the IIRPSS in the CGGA cohort. Distribution of IRS, survival status and Kaplan–Meier survival curves, respectively.

The optimal cutoff values of the 22 immune infiltration cells were displayed in Table S1. LASSO Cox regression analysis was used to construct IIRPSS in the training set (Figures 1A,B). Ultimately, 17 types of immune cells were selected in this scoring system, and the coefficient in the calculation formula for calculating the IRS can be also found in Table S1. The patients were divided into immune high- and low-risk groups based on the optimal IRS 2.626 (Figure 1C) that was founded by “Cutoff Finder,” and their survival statuses were distributed in Figure 1D. Moreover, patients in the high-risk immune group showed a significantly shorter overall survival than the low-risk immune group (Figure 1E, *p* = 1e-10).

To further validate the robustness of the IIRPSS, we applied the same formula and IRS cutoff value to the CGGA validation set. Similarly, every patient was ranked by their IRS (Figure 1F) and assigned to different immune risk groups based on the cutoff IRS. Their survival time and statuses were detailed in Figure 1G. Meanwhile, the K-M curve revealed that the patients in low-risk immune group showed a longer overall survival compared with those in high-risk immune group (Figure 1H, *p* = 0.005).

Additionally, we also demonstrated that the IRS was an independent prognostic factor in GBM by using univariate and multivariate Cox regression analysis in training and validation cohorts (Table 2). Considering the classification of GBM by the world health organization (WHO) in 2016, including GBM IDH-wildtype, GBM IDH-mutant and GBM NOS representing a diagnosis that lacks full IDH evaluation (22). Therefore, the clinical variables (including age and gender), as well as the genetic variable (IDH status), were served as covariates in Cox regression analyses. Moreover, there were only 3 GBM NOS samples in the CGGA cohort, and thus, they were eliminated in the analysis to minimize statistical bias.

**Table 2:** Univariate and multivariate Cox regression analysis in training and validation cohorts.

**Characteristics**		**Univariate**		**Multivariate**	
		**HR (95%CI)**	***p*-value**	**HR (95%CI)**	***p*-value**
TCGA	Age	2.861 (1.956–4.184)	**5.95e-08**	1.837 (1.216-2.776)	**0.004**
	Gender	1.326 (0.976–1.801)	0.071	1.164 (0.852-1.591)	0.341
	IDH mutant	0.257 (0.111–0.591)	**1.39e-03**	0.442 (0.186-1.053)	0.065
	IDH NOS	0.598 (0.442–0.808)	**8.74e-04**	0.718 (0.528-0.976)	**0.034**
	IRS	2.718 (2.125–3.477)	**1.73e-15**	2.322 (1.797-3.001)	**1.21e-10**
CGGA	Age	1.981 (1.098–3.574)	**0.023**	1.554 (0.814-2.967)	0.182
	Gender	0.704 (0.406–1.221)	0.211	0.840 (0.472-1.493)	0.552
	IDH status	0.549 (0.277–1.085)	0.084	0.799 (0.368-1.733)	0.570
	IRS	2.047 (1.324–3.166)	**1.28e-03**	1.798 (1.137-2.843)	**0.012**

Age was defined as 1, ≤45, 2, >45; gender was defined as 0, female, 1, male; status of IDH was given a value of 0, wildtype; 1, mutant; 2, NOS in Cox regression analysis; HR, hazard ratio; CI, confidence interval.

*Bold values means p < 0.05, which represents statistically significant*.

### Nomogram Construction

**Figure 2:**
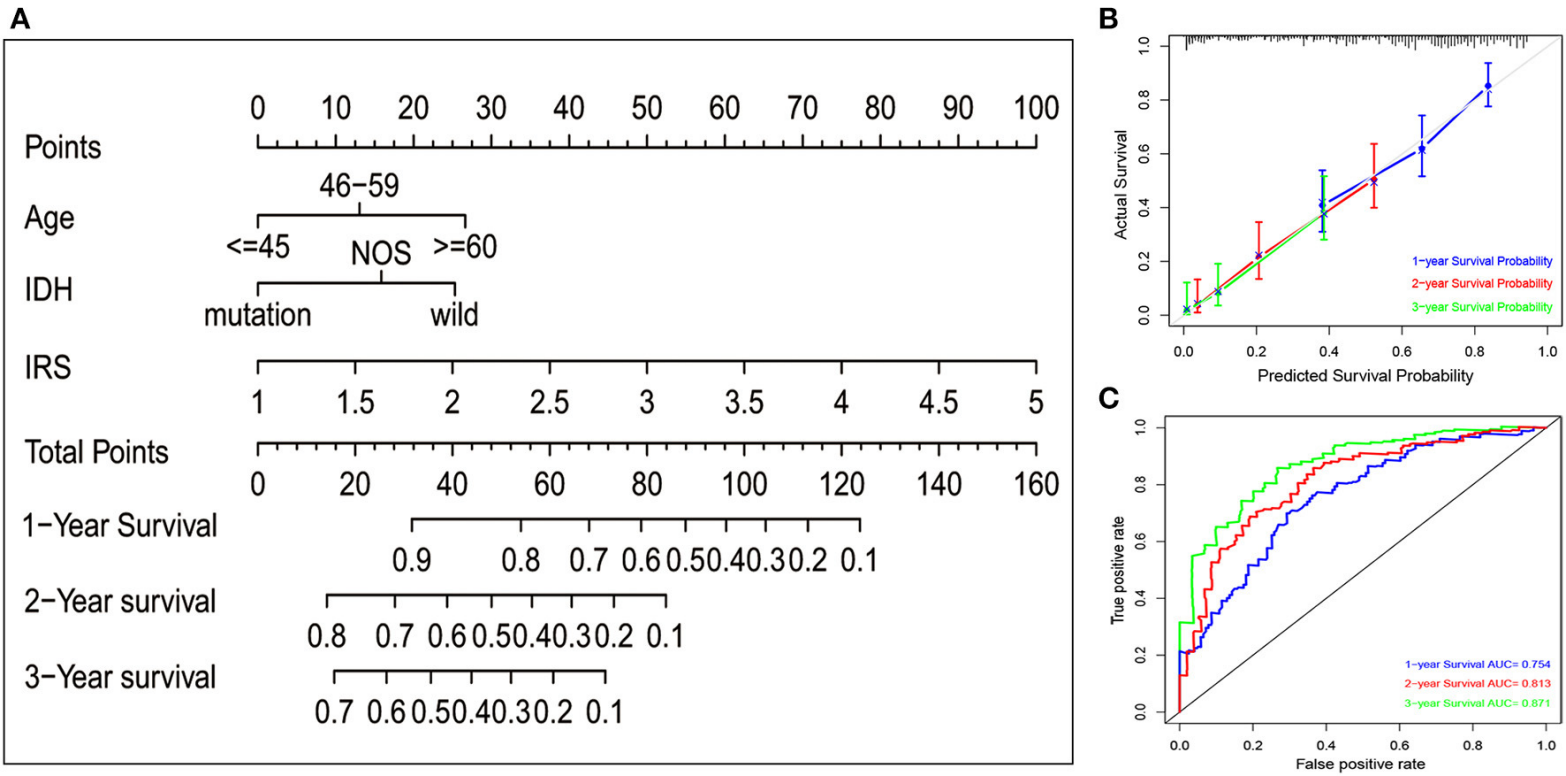
Nomogram construction in TCGA set. **(A)** A nomogram to quantitatively predict 1-, 2-, and 3-year survival for GBM patients based on IRS, clinical and molecular parameters. **(B)** Calibration curves of the nomogram for showing the consistency between predicted and actual survival. **(C)** A series of time-dependent ROC curves for assessing the performance of the prediction nomogram.

To quantitatively predict the prognosis of GBM patients, we constructed a nomogram in TCGA cohort (Figure 2A) that integrated variables showing prognostic trends in Cox analyses. It revealed that the IRS was the leading factor for predicting nomogram, other factors including IDH status showed inferior impact. The calibration plots (Figure 2B) presented a better performance, more importantly, the ROC curve also showed a satisfactory prediction sensitivity and specificity with its 1-year predicting AUC = 0.754, 2-year predicting AUC = 0.813, 3-year predicting AUC = 0.871 (Figure 2C).

### Preliminary Exploration of Immune Microenvironment Based on the IIRPSS

**Figure 3:**
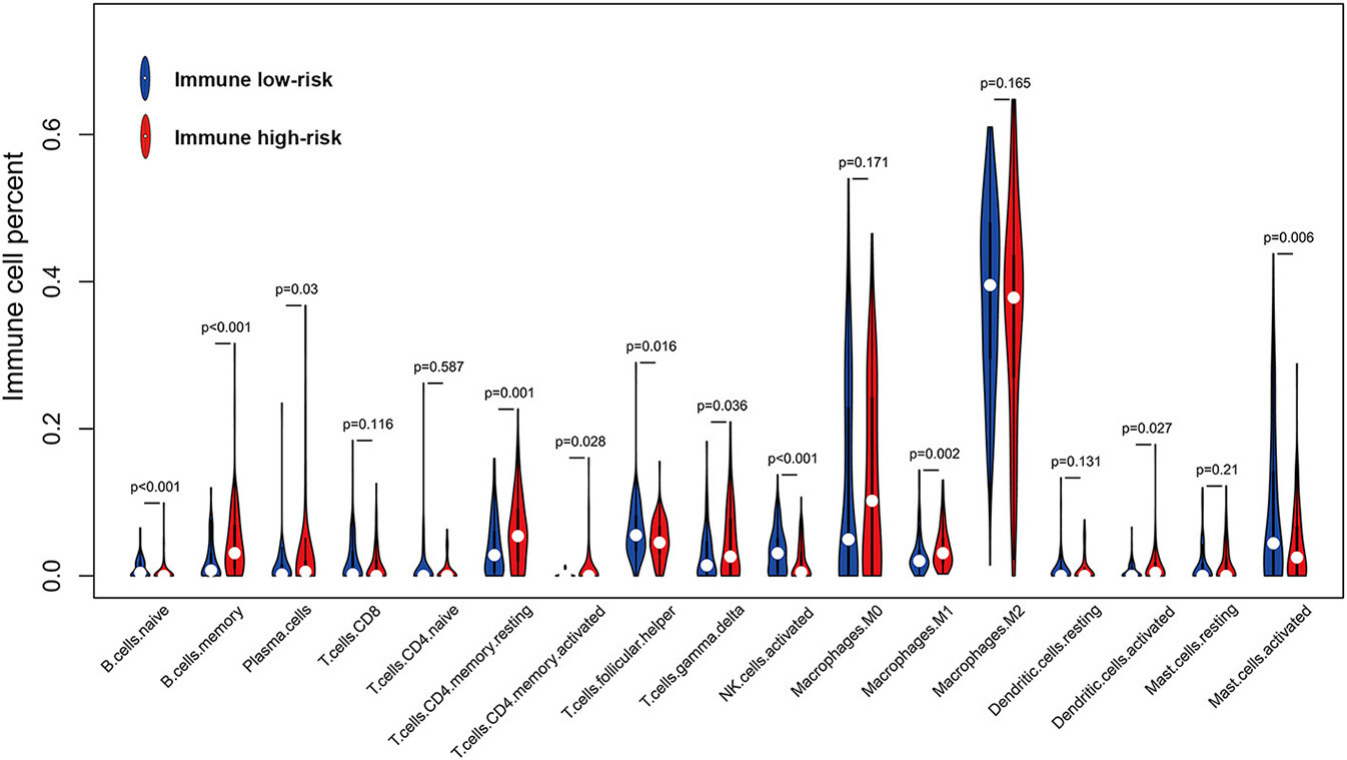
Violin plot for comparing the fractions of 17 types of immune cells included in the IIRPSS between immune low- and high-risk groups in TCGA.

To better understand the immune infiltration microenvironment in GBM, we first investigated the composition of the 17 types of immune cells selected in the IIRPSS construction between different immune risk groups in TCGA cohort. The relative proportion of immune cells derived from CIBERSORT was used for this purpose. As showed in Figure 3, the fractions of immune-infiltrating cells varied obviously, M0 macrophages and M2 macrophages accounted for the majority but no difference was found between groups. Besides, there were 11 types of immune cells significantly differently infiltrated in different immune risk groups. Compared with low-risk immune group, naïve B cells, follicular helper T cells, activated Natural Killer (NK) cells, and activated mast cells were the infiltration is significantly lower in high-risk group, while, memory B cells, plasma cells, resting CD4+ memory T cells, activated CD4+ memory T cells, gamma delta T cells, M1 macrophages and activated dendritic cells were significantly high-infiltrated. Moreover, there were no significant infiltrations of other 4 immune-infiltrating cells observed including CD8+ T cells, CD4+ naïve T cells, resting dendritic cells, and resting mast cells.

**Figure 4:**
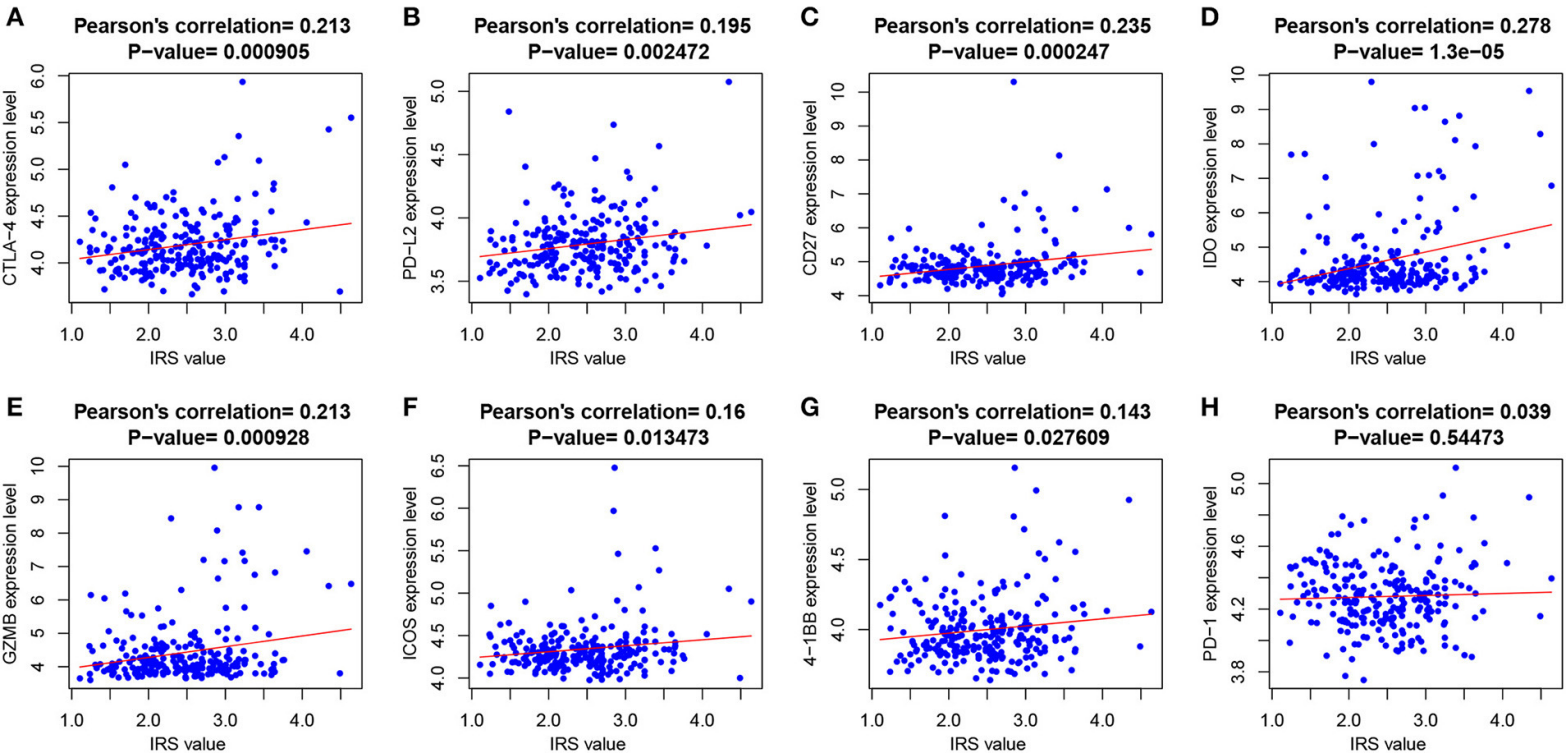
The correlations between IRS and **(A)** CTLA-4; **(B)** PD-L2; **(C)** CD21; **(D)** IDO; **(E)** GZMB; **(F)** ICOS; **(G)** 4-1BB; and **(H)** PD-1.

At the same time, we also analyzed the relationships between immune checkpoint expression and the IRS. The Pearson's correlation analysis (Figure 4) revealed that IRS value was significantly and positively correlated with several immune checkpoints expression, including CTLA-4 (*p* = 9.05e-04), PD-L2 (*p* = 2.47e-03), CD27 (*p* = 2.47e-04), IDO (*p* = 1.3e-05), GZMB (*p* = 9.28e-04), ICOS (*p* = 0.013), and 4-1BB (*p* = 0.028). PD-1 was also positively correlated with IRS but without significance.

**Figure 5:**
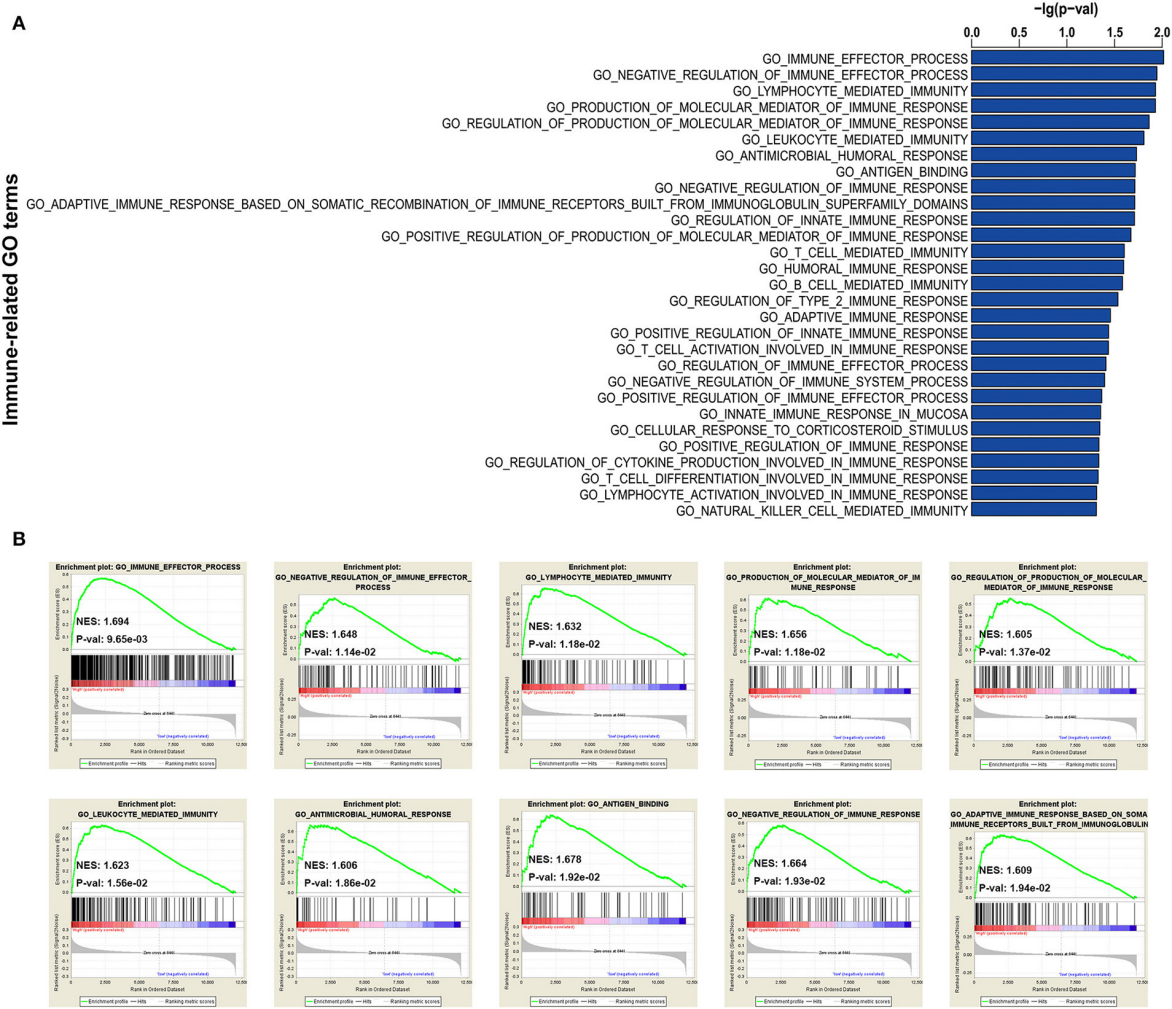
GSEA for comparing immune-related GO terms between immune low- and high-risk groups. **(A)** Total of 29 immune-related GO terms significantly enriched in high-risk immune group. **(B)** The visualization of the top 10 enrichments in high-risk immune group.

In addition, to further explore the immune status between immune high- and low-risk phenotypes, immune-related functional annotation was performed by GSEA. The results suggested that there were 29 immune-related gene ontology terms significantly enriched in high-risk immune group (Figure 5A), but none enriched in low-risk immune group. The top 10 items were further visualized in Figure 5B.

## Discussion

GBM is a brain tumor characterized by highly intratumoral heterogeneity and intratumoral heterogeneity (10), which leads to an embarrassing situation that only a subset patient acquire therapeutic efficacy after checkpoint blockade immunotherapy. Facing this challenge, it may be a feasible strategy to stratify patients and find those who are most likely to benefit from immunotherapy. However, traditional methods for detecting immune-infiltrating cells, such as flow cytometry or immunohistochemistry (17), have the defection of not being able to comprehensively assessing different immune cells or discriminate the closely related cell subpopulations. These methods are heavily limited by the number of fluorescent channels and immunophenotypic markers available (16). CIBERSORT, a bioinformatics tool, was known as the most accurate method available (17) to integrate transcriptomics profiles and analyze the large-scale immune landscape.

Thus, in the present study, we first used the novel CIBERSORT algorithm to analysis 22 immune cell subsets landscape in patients with GBM based on TCGA transcriptomics data and further construct an IIRPSS by LASSO Cox regression analysis. The patients could be successfully classified into different groups according to their IRS, and their prognoses were significantly varied from group to group. Additionally, the performance of this IIRPSS was validated in the CGGA cohort. Moreover, the IRS was demonstrated as an independent prognostic factor by univariate and multivariate Cox analyses. Most important, to predict the survival of patients with GBM more accurately, a predicting nomogram was established on the basis of IRS. Besides, this nomogram also combined patient's age and IDH status. IDH status was the molecular marker in 2016 WHO classification of GBM. Interestingly, the IRS was the dominant factor in the nomogram rather than IDH status, indicating our IIRPSS was superior to IDH status as an excellent prognostic factor.

Considering the importance of immune microenvironment in the progression of GBM and the efficacy of individualized immunotherapy, we preliminarily explored the immune microenvironment based on the IIRPSS. Firstly, the 17 types of immune-infiltrating cells incorporated in the construction of this IIRPSS were explored between different immune risk groups. Here we discussed several immune-infiltrating cells most relevant to anti-GBM. Macrophages were the most majority tumor immune-infiltrating cells in GBM, including M0, M1, and M2 cells. It is generally considered that M0 macrophages are unactivated and without specific function (19). While M0 can differentiate into M1 and M2 under different stimulations, and they, respectively, exhibit inflammatory response against tumor cells and immunosuppressive response promoting tumor cells proliferation and differentiation (23). However, this concept of dual-polarization status is likely to be oversimplified (24) and is strongly debated (25). In addition, there is a great challenge of this cognition that tumor-related macrophages could co-express M1 and M2 biomarkers and a continuously activated macrophage exists in GBM (25). More importantly, the function of macrophage will changes quickly when exposure to GBM, leading to innate and adaptive immune suppression (26). Therefore, reversing the immunosuppressive microenvironment caused by macrophages may be promising therapies in GBM. Previous bioinformatics analysis showed that macrophages play a negative role, while CD8+ T cells play a positive role in survival prediction of GBM patients (27). Pan-cancer analysis also revealed that several T cells, including gamma delta and CD8+ T cells, are broadly favorable prognostic signatures (28). Tumor-infiltrating T cells (29), especially CD8^+^ cytotoxic T lymphocytes, act as critical components of adaptive immunity in attacking tumor cells. However, their low infiltrating levels also indicate the impaired immune function in GBM; while tumor-infiltrating CD4^+^ T cells play the roles of a double-edged sword in tumor-specific immunity. Follicular helper CD4^+^ T cells (30) perform a central role in initiating and activating immune responses against tumor and helping CD8+ T cells function, which may be a reason why it is relatively high in the low-risk immune group. On the contrary, CD4^+^ regulatory T cells inhibit anti-tumor immunity and accelerate tumor progression. Even worse, the majority of CD4^+^ tumor-infiltrating T cells suppress immune response in GBM (29), which may explain the phenomenon that resting and activated CD4^+^ T cells are both higher in high-risk immune group. However, the truth may be much more complicated. Han et al. (29) found that either high CD4^+^ and low CD8^+^ T cells level or low CD4^+^ and high CD8^+^ T cells level correlates with poor prognosis, indicating that appropriate CD8^+^/CD4^+^ T cells ratio is much important to maintain effective anti-tumor immunity and could be therapeutically significant. NK cells (31), as important cellular components of the innate immune system, can directly kill tumor cells without previous activation, and the cytotoxic activity is powerful. Consistent with our findings, Studies revealed that NK cells are a low infiltrated population of immune cells in the GBM tumor microenvironment, but they still have great cytotoxic activity (32). However, Wu et al. found that the lack of NK cells was associated with better prognosis in GBM. Therefore, how the infiltrating NK cells functions in GBM microenvironment still need more studies to eclucidate (33).

The complexity of the immune microenvironment of GBM is a great impediment to successful immunotherapy. Recently, immune checkpoint inhibitors (ICIs) initiated a new era of anti-cancer immunotherapy (34). Therefore, we performed a deeper investigation for exploring the relationships between immune checkpoints and immune risk groups as well as the immune statuses of different groups to look for the patients who may be likely to obtain the maximal benefit from immune checkpoint blockade. Our GSEA findings indicated an obvious enrichment of immune-related GO terms in the high-risk immune group, including innate and adaptive immune responses, which could reflect a more active immune microenvironment in this group. However, several immune checkpoints tended to express higher in the microenvironment. It is a reasonable speculation that the active immune microenvironment is inhibited by relevant immune checkpoint protein-mediated suppression pathways. So, the high-risk immune group patients may be the biggest beneficiaries of ICIs. These results were in accordance with previous studies (35–38). Recently, Han et al. demonstrated that immune checkpoint molecule herpes virus entry mediator (HVEM), also known as tumor necrosis factor receptor (TNFR) superfamily 14 (TNFRSF14), is over expressed and related to poor prognosis in GBM (39). HVEM maybe serve as a new immune-therapeutic target for the treatment of GBM. Considering limited studies have been conducted, further studies are still needed to achieve effective and successful immunotherapy in GBM.

There were some limitations to the present study. First, it was a retrospective study, which may lead to an analytical bias. Therefore, a prospective cohort study is needed to achieve a better fit. Second, genomic landscape analysis revealed some aspects of the immune microenvironment but lacked comprehensive exploration.

In summary, our study used the immune-infiltrating cells to construct an IIRPSS for the first time in GBM, classifying patients into different immune risk groups. In addition, we further established a nomogram to quantitatively predict a patient's survival based on IRS derived from this IIRPSS as well as clinical data. Moreover, we also preliminarily explored the differences in the immune microenvironment between different groups. These findings may provide a guideline to identify candidates most likely to benefit from ICIs and valuable resources to improve personalized immunotherapy of GBM patients.

## Data Availability Statement

The datasets included in this study are available from the UCSC Xena website (https://xena.ucsc.edu/) and the Chinese Glioma Genome Atlas (CGGA) (http://www.cgga.org.cn/index.jsp). More information of TCGA data and CGGA data are below:

dataset ID: TCGA.GBM.sampleMap/HT_HG-U133A
download: https://tcga.xenahubs.net/download/TCGA.GBM.sampleMap/HT_HG-U133A.gz; Full metadata
dataset ID:mRNAseq_693; mRNAseq_325
download http://www.cgga.org.cn/download.jsp.

## Author Contributions

WY conceived and designed the study, analyzed the results, and completed the image visualization. GT wrote the manuscript. All authors participated in the preparation approved the final manuscript.

### Conflict of Interest

The authors declare that the research was conducted in the absence of any commercial or financial relationships that could be construed as a potential conflict of interest.
