# Supplementary material for: Development of an Immune Infiltration-Related Prognostic Scoring System Based on the Genomic Landscape Analysis of Glioblastoma Multiforme
Source: Front Oncol. 2020 Feb 18;10:154. doi: 10.3389/fonc.2020.00154 (PMC7040026; doi:10.3389/fonc.2020.00154)
Supplement: Supplementary file 1 [file Table_1.DOCX]

**Table S1** Cut-off value for immune cell fractions and the coefficient in calculation formula for calculating the immune risk score

| **Immune Cell** | **Optimal cut-off value** | **Coefficient** |
| --- | --- | --- |
| B cells naive | 0.01052 | -0.61598 |
| B cells memory | 0.09311 | 0.85128 |
| Plasma cells | 0.01378 | -0.36765 |
| T cells CD8 | 0.05257 | -0.03378 |
| T cells CD4 naive | 0.0006786 | 0.13883 |
| T cells CD4 memory resting | 0.04164 | 0.44011 |
| T cells CD4 memory activated | 0.0167 | 1.16166 |
| T cells follicular helper | 0.09769 | -0.30427 |
| T cells regulatory | 0.00497 |  |
| T cells gamma delta | 0.04704 | 0.21192 |
| NK cells resting | 0.05415 |  |
| NK cells activated | 0.006136 | -0.24314 |
| Monocytes | 0.003721 |  |
| Macrophages M0 | 0.09083 | 0.31427 |
| Macrophages M1 | 0.005488 | 0.39254 |
| Macrophages M2 | 0.6104 | 0.92313 |
| Dendritic cells resting | 0.005164 | -0.36496 |
| Dendritic cells activated | 0.02611 | 0.35847 |
| Mast cells resting | 0.08652 | -0.54988 |
| Mast cells activated | 0.1447 | -0.21077 |
| Eosinophils | 0.01845 |  |
| Neutrophils | 0.01284 |  |
